# High Genetic Diversity and Antimicrobial Resistance in *Escherichia coli* Highlight *Arapaima gigas* (Pisces: Arapaimidae) as a Reservoir of Quinolone-Resistant Strains in Brazilian Amazon Rivers

**DOI:** 10.3390/microorganisms10040808

**Published:** 2022-04-13

**Authors:** Luciana Sampaio Lima, Aldo Aparecido Proietti-Junior, Yan Corrêa Rodrigues, Marcelo Cleyton da Silva Vieira, Luana Nepomuceno Gondim Costa Lima, Cintya de Oliveira Souza, Verônica Dias Gonçalves, Marcelo de Oliveira Lima, Dália dos Prazeres Rodrigues, Karla Valéria Batista Lima

**Affiliations:** 1 Faculty of Pharmaceutical Sciences, Federal University of Amapá (UNIFAP), Macapá 68903-419, AP, Brazil; aldo.proietti@unifap.br; 2 Bacteriology and Mycology Section, Evandro Chagas Institute (SABMI/IEC), Health Surveillance Secretariat, Ministry of Health, Ananindeua 67030-000, PA, Brazil; yan.13@hotmail.com (Y.C.R.); marcelocleiton14@hotmail.com (M.C.d.S.V.); luanalima@iec.gov.br (L.N.G.C.L.); cintyaoliveira@iec.gov.br (C.d.O.S.); 3 Oswaldo Cruz Institute, Oswaldo Cruz Foundation, Pavilhão Rocha Lima, Enterobacteria Laboratory, Rio de Janeiro 21040-900, RJ, Brazil; vegonc@bol.com.br (V.D.G.); dalia@ioc.fiocruz.br (D.d.P.R.); 4 Environment Section, Evandro Chagas Institute (SAMAM/IEC), Health Surveillance Secretariat, Ministry of Health, Ananindeua 67030-000, PA, Brazil; marcelolima@iec.gov.br

**Keywords:** quinolone-resistance, antimicrobial resistance, ESBLs, PMQR, genetic diversity, molecular epidemiology, food-producing animals, pirarucu, Amazon region

## Abstract

The increasing prevalence of multi-drug resistant (MDR) *Escherichia coli* in distinct ecological niches, comprising water sources and food-producing animals, such as fish species, has been widely reported. In the present study, quinolone-resistant *E. coli* isolates from *Arapirama gigas*, a major fish species in the Brazilian Amazon rivers and fish farms, were characterized regarding their antimicrobial susceptibility, virulence, and genetic diversity. A total of forty (40) specimens of *A. gigas*, including 20 farmed and 20 wild fish, were included. Thirty-four quinolone-resistant *E. coli* isolates were phenotypically tested by broth microdilution, while resistance and virulence genes were detected by PCR. Molecular epidemiology and genetic relatedness were analyzed by MLST and PFGE typing. The majority of isolates were classified as MDR and detected harboring *bla*_CTX-M_, *qnrA* and *qnrB* genes. Enterotoxigenic *E. coli* pathotype (ETEC) isolates were presented in low prevalence among farmed animals. MLST and PFGE genotyping revealed a wide genetic background, including the detection of internationally spread clones. The obtained data point out *A. gigas* as a reservoir in Brazilian Amazon aquatic ecosystems and warns of the interference of AMR strains in wildlife and environmental matrices.

## 1. Introduction

Antimicrobial-resistant (AMR) bacteria are constantly reported in water systems as this type of environment receives most of the hospital, domestic and industrial waste without proper treatment, leading to the existence of different and complex AMR determinants pools, encompassing both terrestrial and aquatic ecosystems [1,2,3]. Furthermore, the increasing detection of several multidrug-resistant (MDR) bacterial species in fish and other food-producing animals has been widely reported and has raised concern among authorities regarding the transmission to humans through food chains, as well as survival and mortality aspects in animal farming [3,4,5,6,7,8].

Although typically part of the normal microbiota of warm-blooded animals, *Escherichia coli* is also abundant in water systems and considered a resistance-indicator species of major epidemiological relevance due to its ability to transfer genes to other pathogenic bacteria, playing a significant role in the dissemination of AMR genetic determinants [7,9,10]. In addition, *E. coli* isolates can be classified as commensal or pathogenic, where pathogenic isolates related to intestinal diseases in animals, and can be subdivided based on the detection of genes: enterotoxigenic—ETEC (*lt* or *st*), enterohemorrhagic—EHEC/STEC (*eae*A and at least one *sxt*), enteropathogenic—EPEC (*aea*A), possibly diffusely adherent—DAEC (fimbrial adhesins *afa* and *dr*), entero-invasive—EIEC (*ial*), entero-aggregating—EAEC (*eagg*), adherent-invasive—AIEC (*fim*H) and enteroaggregative hemorrhagic *E. coli*—EAHEC (*stx*2) [11,12].

Among the several antimicrobial classes, quinolones and beta-lactams are often used in the human and veterinary fields. Previously reported data demonstrated resistance against those antimicrobials in water and fish *E. coli* isolates along with the plasmid-mediated quinolone-resistance determinants (PMQR) and extended spectrum β-lactamases (ESBLs), demonstrating the importance of such bacterial species as reservoirs and in the spread of AMR determinants to humans [2,7,13,14,15,16,17,18,19,20].

In the Brazilian Amazon region, the *Arapaima gigas* species, also known as “pirarucu or the giant of the Amazon” is a fish of major ecological, economic, and cultural importance, occupying a prominent place in Brazilian aquaculture [5,21]. The production of this species is concentrated in the Amazon region and reaches 11,763 tons annually, the seventh-largest species in the volume of production in Brazil [22]. Considering that fish species may pose as resistance reservoirs and the importance of *A. gigas* farming in the Amazon region, the present study reports the resistance and virulence features, as well as the genetic diversity of *E. coli* isolated from the intestinal content of wild and farmed *A. gigas* in the far north of the Brazilian Amazon. 

## 2. Materials and Methods

### 2.1. Study Setting and Sample Collection

**Figure 1:**
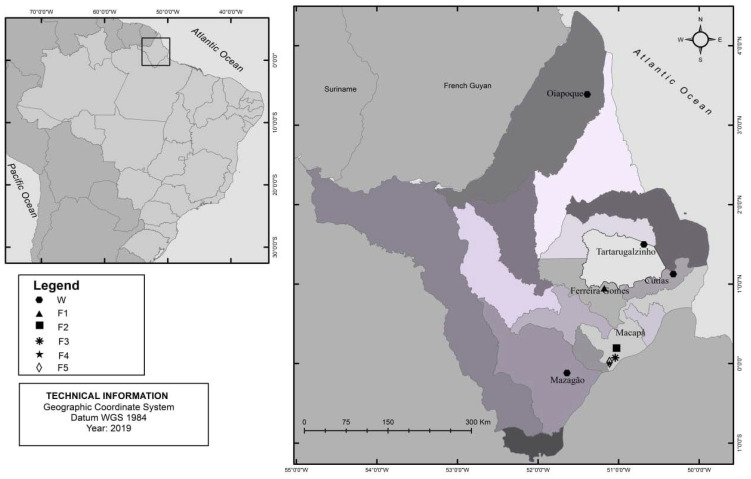
Collection points of farmed and wild *Arapaima gigas* specimens, State of Macapá, Brazilian Amazon. W: wild, F: Fish farm.

The collection of *A. gigas* specimens was performed in five fish farms and five rivers in the state of Amapá, in the far north of the Brazilian Amazon (Figure 1). The five fish farms (F1—0°11′41.7″ N 51°01′29.6″ W, F2—0°04′23.8″ N 51°02′28″ W, F3—0°00′26.4″ N 51°06′46″ W, F4—0°02′34.9″ S 51°07′50.0″ W, and F5—0°00′24.2″ S 51°06′15.6″ W) were selected based on information provided by the Amapá State Fisheries Agency (PESCAP). Wild *A. gigas* were recovered in the municipalities of Mazagão (MZG—0°06′56.8″ S 51°38′21.9″ W), Cutias (CUT—1°07′35.6″ N 50°19′13″ W), Ferreira Gomes (FG—0°56′53.4″ N 51°10′39.2″ W), Oiapoque (OPQ—3°34′19.3″ N 51°23′15.3″ W) and Tartarugalzinho (TT—1°29′58.7″ N 50°41′07.4″ W). A total of forty (40) specimens of *A. gigas* were collected between November (2017) and June (2019), including 20 farmed and 20 wild fish.

### 2.2. Bacterial Isolates and Antimicrobial Susceptibility Testing

The *A. gigas* specimens were individually transported to the laboratory in sterile plastic bags within a water tank in an isothermal box and submitted to cryoanesthesia, followed by euthanasia by the spinal cord section. Samples of four grams of *A. gigas* intestinal content were incubated overnight at 37 °C in 36 mL of MacConkey (BD) broth supplemented with ciprofloxacin (CIP, 10 µg/mL), for selection of quinolone-resistant *E. coli* isolates. One hundred µL (100 µL) aliquots of this culture were inoculated on MacConkey agar plates and incubated under the same conditions. Isolated colonies were phenotypically identified on the automated VITEK 2^®^ system equipment (bioMérieux, Marcy I’Etoile, France).

The antimicrobial susceptibility testing (AST) determined the minimum inhibitory concentration (MIC) by broth microdilution using the AST 239 card on the automated VITEK 2^®^ system equipment (bioMérieux, Marcy I’Etoile, France) for the following antimicrobials: ampicillin (AMP), ampicillin/sulbactam (SAM), piperacillin/tazobactam (TZP), cefuroxime (CXM), cefuroxime axetil (CXM), cefoxitin (FOX), ceftazidime (CAZ), ceftriaxone (CRO), cefepime (FEP), ertapenem (ETP), imipenem (IMP), meropenem (MEM), amikacin (AMK), gentamicin (GEN), ciprofloxacin (CIP), tigecycline (TGC), and colistin (COL). Results were interpreted according to the guidelines and breakpoints of the Clinical and Laboratory Standards Institute [23]. The reference strain *E. coli* ATCC 25922 was used as a quality control. In addition, *E. coli* isolates were phenotypically categorized as MDR if resistant to ≥1 drug in ≥3 antimicrobial categories, according to criteria proposed by [24].The Multi-Antibiotic Resistance Index (MAR) was determined by means of the following formula: x/y, where ‘x’ indicates the number antibiotics for which the isolate was resistant, and ‘y’ indicates the number of antibiotics tested against the isolate. Results higher than 0.2 indicate that the microorganism is a potential reservoir of resistance determinants [25].

### 2.3. Molecular Detection of Antimicrobial Resistance and Virulence Markers

Bacterial DNA was extracted using the PureLink^®^ commercial kit (Invitrogen), according to the manufacturer’s guidelines. The molecular detection of quinolone and beta-lactam-resistance genes was performed by PCR on Veriti thermocycler (Applied Biosystems, Foster City, CA, USA), using previously describer primers [26,27,28,29,30,31,32]. Each PCR reaction was prepared with a total volume of 20 µL, including 2.5 µL of 10× PCR buffer (New England BioLabs, Ipswich, MA, USA), 5 pmol of each primer (MWG- Biotech AG, Ebersberg, Germany), deoxynucleoside triphosphates at a concentration of 200 µM (Promega, Madison, WI, USA), 1U Taq DNA polymerase (New England BioLabs), and 50 ng of genomic DNA.

The virulence genes were detected by multiplex PCR, applying a slightly modified protocol by Omar et al. [33]. Each reaction was prepared with a total volume of 20 µL, 2.5 µL of 10× PCR buffer (New England BioLabs, Ipswich, MA, USA), 5 pmol of each primer (MWG-Biotech AG, Ebersberg, Germany), deoxynucleoside triphosphates at a concentration of 200 µM (Promega), 1U Taq DNA polymerase (New England BioLabs) and 50 ng of genomic DNA. Amplifications were performed on Veriti thermocycler (Applied Biosystems, Foster City, CA, USA) under the following conditions: 95 °C for 15 min; followed by 30 cycles at 94 °C for 45 s, 55 °C for 45 s, 68 °C for 2 min and final extension at 72 °C for 5 min. PCR products were subjected to electrophoresis on 2% agarose gel (Sigma) stained with ethidium bromide and amplicons were visualized in the Image Quant transilluminator.

### 2.4. Molecular Typing by Multilocus Sequence Typing (MLST)

MLST genotyping was performed according to the protocol of [34]. The seven housekeeping genes—*adk* (adenylate kinase), *fumC* (fumarate hydratase), *gyrB* (DNA gyrase), *icd* (isopropylmalate dehydrogenase), *mdh* (malate dehydrogenase), *purA* (adenylosuccinate dehydrogenase), and *recA* (ATP/GTP binding motif) —were amplified for each sample by PCR on the Veriti thermocycler (Applied Biosystems, Foster City, CA, USA). Reaction products were sequenced in both strands with previously published primers on the ABI Prism 3130 Genetic Analyzer Platform (Applied Biosystems, Foster City, CA, USA). The results obtained were analyzed according to the PubMLST database (https://pubmlst.org/escherichia/, accessed on 21 February 2022) for determination of sequence types (STs).

### 2.5. Molecular Typing by Pulsifield Gel Eletroforese (PFGE)

The genetic relationship of *E. coli* isolates was analyzed by Pulsed Field Gel Electrophoresis (PFGE) with XbaI (Roche) DNA digestion, according to the PulseNet protocol [35]. The preparation of the inoculum was initially obtained by sowing the microorganism by depletion in Nutrient Agar and subsequent incubation (24 h/35 °C) to obtain isolated colonies, from which suspensions were prepared in 4 mL of buffer for cell suspension with the aid of a sterile swab until turbidity between 8 and 10% absorbance was obtained in the 610 nm wavelength spectrophotometer (corresponding 3 × 10^8^ CFU/mL).

Agarose plugs were produced from 200 μL aliquots of each bacterial suspension and transferred to sterile polypropylene R tubes, to which 10 μL of Proteinase K (20 mg/mL), were added to 200 μL of Seakem Gold agarose 1% in TE buffer. Under constant homogenization and dispensing in 46 molds at 50 °C, small blocks of agarose gel containing genomic DNA (2 blocks per sample) were obtained. After proper assembly of the digested blocks in the comb, the agarose was introduced in the horizontal gel holder, where it remained for 30 min at room temperature for solidification. The enzyme used to digest the bacterial DNA was XbaI, which generated fragments of 50–300 kpb, with a 5 “-30”/23 h. The fragments generated by the enzymatic restriction were visualized in UV light in a photo-documentation device for further analysis. Finally, the agarose-soaked bacterial DNA was digested with restriction enzyme XbaI at 37 °C for 4 h. In addition, electrophoresis was performed on 1% agarose gel with 0.5× Tris-borate-EDTA buffer.

PFGE standards and computer-assisted cluster analysis were visually analyzed using BioNumerics^®^ software, version 7.6 (Applied Maths, SintMartens-Lantem, Bélgica). The similarity between the pairs was obtained by analysis based on a dendrogram, calculated with the data coefficient and the unweighted arithmetic mean pairs (UPGMA) method. *Salmonella enterica* serotype Braenderup, strain H9812 (ATCC BAA 664) was used as the molecular size standard.

### 2.6. Statistical Analysis

Statistical analyses to compare isolates’ resistance profiles from wild and captive groups were performed using the binomial proportion test. Values of *p* ≤ 0.05 were considered statistically significant. All analyzes were performed using the statistical software BioEstat^®^ 5.3 [36].

### 2.7. Ethical Considerations

This study was evaluated by the Animal Research Ethics Committee of the Federal University of Acre (CEUA/UFAC), process Nº. 23107.009564/2014-29 (approval date: 10 July 2014), in compliance with the Brazilian regulation Law Nº 11,794 of 2008, which deals with animal experimentation in Brazil.

## 3. Results

### 3.1. Antimicrobial Resistance and Virulence Features

A total of 34 quinolone-resistant *E. coli* were recovered from 16 farmed and 18 wild fish. AST revealed that the majority of *E. coli* isolates were MDR (21, 61.7%), presenting non-susceptible to AMP (*n* = 27, 79.4%), followed by CXM (*n* = 24, 70.6%), and SAM (*n* = 22, 64.7%). Oppositely, all tested isolates were susceptible to ETP, IMP, MEM, AMK, GEN, TGC, and COL. The MAR index differed among isolates group, with a MAR index of 0.28 and 0.2 observed on *E. coli* from wild and farmed fish, respectively. The *E. coli* isolates recovered from wild fish demonstrated a significantly higher frequency of nonsusceptibility phenotypes (*p* = 0.005) (Table 1). The molecular detection of AMR markers revealed 15 (44.1%) isolates harboring the *bla*_CTX-M_ gene, including 10 (55.5%) from wild and five (31.2%) from farmed fish. In addition, PMQR genes were mostly detected among farmed fish, including *qnrA* (4–25.0%) and *qnrB* (2–12.5%), while one isolated from wild fish at CUT was detected harboring the *qnrB* gene (Table 2).

Concerning the presence of genes related to virulence investigated by PCR, only three strains (8.82%) revealed the presence of the *st* gene responsible for the enterotoxigenic *E. coli* pathotype (ETEC), all from farmed animals at F3.

### 3.2. Molecular Typing by MLST and PFGE

**Figure 2:**
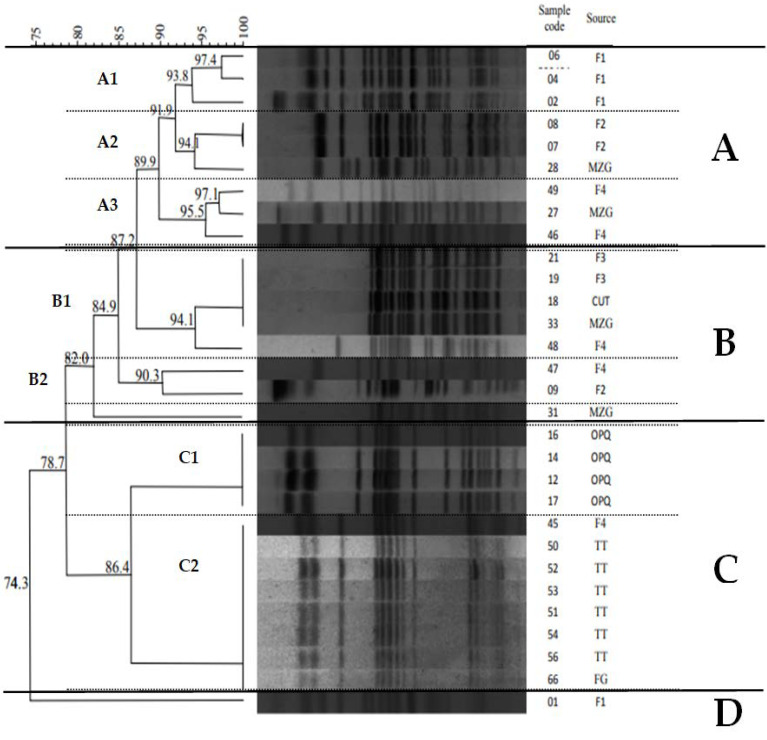
PFGE dendogram of demonstrating the genetic relatedness of *E. coli* isolated from *A. gigas* in the Brazilian Amazon. F: Fish farm, CUT: Cutias; FG: Ferreira Gomes, MZG: Mazagão; OPQ: Oiapoque; TT: Tartarugalzinho; (**A**–**D**): clusters; (**A1**–**C2**): subclusters.

Thirty-two (32) and 30 isolates were genotyped by MLST and PFGE, respectively, revealing a wide genetic background. MLST analysis demonstrated the presence of eleven distinct types, including: ST155 (3, 9.4%), ST224 (4, 12.5%), ST226 (1, 3.0%), ST448 (4, 12.5%), ST949 (1, 3.0%), ST1196 (1, 3.0%), ST1249 (3, 9.4%), ST1431 (2, 6.25%), ST4380 (1, 3.0%), ST4482 (1, 3.0%) and ST7973 (8, 25.0%). The *bla*_CTX-M_ gene associated with several *E. coli* genotypes: ST448, ST155, ST4482, ST226, ST1431 and ST7973; while *qnrA* gene with ST448, ST1249 and ST155, and *qnrB* with ST448. Worryingly, the ST448 was detected at F1 among isolates harboring the *bla*_CTX-M_ and *qnr* genes (Table 2). At F3, enterotoxigenic *E. coli* isolates were suggested to be genetically related by MLST and PFGE (ST155 and subcluster B1) (Table 2 and Figure 2).

Through the PFGE technique, 16 pulsotypes were identified, divided into four distinct clusters and seven subclusters (Figure 2 and Table 2). Interestingly, *E. coli* isolates presenting similar genetic signatures by PFGE were detected in distinct locations: strains of subcluster A2, including ST1249 and ST949, were associated with wild and farmed fish in F2 and MZG; strains of subcluster A3, including ST1431 and ST4380, were associated with wild and farmed fish in F4 and MZG; strains of subcluster B1, including ST155 and ST1196, were associated with wild and farmed fish in F3, F4, CUT and MZG; strains of subcluster B2, including S224 and ST7973, were associated with farmed fish in F2 and F4; strains belonging to subcluster C2, which also included ST7973 and ST226, were associated with wild and farmed fish in TT, FG and F4 locations; ST224, ST226, ST1196 and ST1431 were associated with farmed fish at F4 location; ST949, ST4380 and ST4482 were associated with wild fish at MZG location (Table 2 and Figure 2). On the other hand, genetic related strains were also found among isolates from the same location: strains belonging to subcluster A1, including ST448, were associated with farmed fish in F1, while strains from subcluster C1, including ST224, were associated with wild fish in OPQ (Figure 2 and Table 2).

## 4. Discussion

*A. gigas* is a native fish that occupies the top of the aquatic food chain in the Brazilian Amazon, reaching weights close to 125 kg, highlighting its elevated economic value; therefore, it can be considered a critical epidemiological indicator of environmental resistance, and may also act as a possible source of dispersion of AMR bacteria to humans and other wild and farmed animals. Gram-negative bacteria in the water are usually related to fecal contamination, showing how vital are the tracking process and characterization of *E. coli* isolates in the environment. *E. coli* is one of the major bacterial species capable of transferring/exchanging genetic information with other bacterial groups, contributing to the dissemination of AMR to distinct environments [37]. Different fish species have been found carrying *E. coli* resistance genes related to essential antimicrobials, such as quinolones and beta-lactams [7,38,39,40]. In the present study, this bacterial species was found among farmed and wild *A. gigas*, highlighting the geographical aspects of the presence of resistant isolates in fish in the Amazon region, and suggests an impact of human and antimicrobials interference in different ecological niches.

Data from the Netherlands evaluating ESBL-producing *E. coli* from water samples demonstrated that approximately 84% of this bacterial species exhibits resistance in up to three drug classes, including beta-lactams, tetracyclines, and aminoglycosides [38]. In addition, *E. coli* was the only species isolated in different fishes (*Oncorhynchus mykiss*, *Squalius cephalus* and *Cyprinus carpio*) from the farms and local markets in Turkey, including quinolone-resistant and MDR isolates [39]. ESBL-producing enterobacteria represent a significant role in health infections, increasing hospitalization time, morbidity, and mortality rates. Several bacterial species harboring *bla*_CTX-M_ gene have become a major burden in clinical settings [41,42,43,44,45]. Other studies with ESBL-producing *E. coli* conducted in North America, Europe, South America, and Africa have shown similar high frequency results of CTX-M group strains showing simultaneous resistance to cephalosporins and quinolones [2,15,38,42,46]. Information regarding *E. coli* strains and circulation of resistance genes is produced by the present study, and this data is epidemiologically crucial to establish measures aiming to reduce the resistance burden.

In recent years, many studies have shown a worldwide exponential increase in quinolone and beta-lactam drug resistance in animal *E. coli* isolates, including fish, primarily associated with the presence of PMQR and ESBLs, demonstrating the importance of these reservoirs in the spread of antimicrobial resistance determinants to humans [9,14,15,16,39,40]. PMQR contribute with the selection of highly resistance pathogens, posing a threat to treatment, and act as a latent reservoir for disseminating these attributes to other aquatic organisms [47,48]. Among the surveyed PMQR, the *qnr*A (14.7%) gene was the most frequent in samples from farmed and wild fish, while the *qnr*B was detected in only 5.8%. Such determinants were previously reported among Brazilian clinical *E. coli* isolates, while the *qnr*S and *aac*(6-)-Ib-cr genes, frequently detected in Brazil and other Latin American countries, were not observed in this study [49,50,51].

MLST is a globally applied and reliable tool for investigating the population structure of bacteria such as *E. coli*, providing data on lineages, resistance and virulence features that are often involved in human and animal diseases, in addition to those isolates which are not typable by the standard PFGE method due to DNA methylation [52,53,54]. MLST genotyping revealed wide genetic diversity also supported by the PFGE analysis, with the presence of 11 different STs recognized for harboring distinct AMR markers at several locations, in addition to rarely detected clones. Data regarding MLST genotypes of *E. coli* from the Brazilian Amazon is scarce; therefore, results from the present report are also valuable for epidemiological surveillance of international clones of clinical and veterinary importance.

ST7973 was the most frequently detected clone (23.4%) and was associated with the carriage of the *bla*_CTX-M_ among genetically related *E. coli* isolates from wild fish at TT and FG (subcluster C2). This is the first report of this clone in Brazil. Concerningly, this ST has been previously associated with *mcr-1* gene in an abscess isolate in Venezuela and raw turkey meat in Poland, underscoring this genotype role in the threat to last-resort antibiotics [55,56,57].

Several studies demonstrated the typical relation of ST448 with ESBL-producing and quinolone-resistant *E. coli*, among which were those conducted with a veterinary approach in Spain and the Netherlands which describe several isolates harboring the *bla*_CTX-M_ gene [58,59]. In clinical settings, this genotype has been reported in Brazil among CMY-producing isolates, and in other locations, such as: among ESBL-producing *E. coli* in Dutch patients with gastrointestinal complaints; in a wound sample in Nigeria; in a urinary infection isolate exhibiting co-resistance to aminoglycosides and oxyiminocephalosporins in the USA, and recently, with an IncX3 NDM-5-producing plasmid isolate in Mali [60,61,62,63,64]. n the present report, *E. coli* strains belonging to ST448 were associated with different AMR markers, including *bla*_CTX-M_, *qnrA* and *qnrB* genes, highlighting its transference capability of genetic traits and high-risk clone potential of causing outbreaks and dissemination in both clinical and veterinary settings.

*E. coli* ST155 is a major reported lineage among poultry from Africa, Europe, and South America, and water sources in India and Japan, and demonstrated as being critically responsible for the spreading of highly drug resistant strains from animal and food-producing sources to humans [65,66,67,68,69,70,71,72]. Molecular typing data obtained suggest the dissemination, among wild and farmed fish (F3 and CUT), of genetically related enterotoxigenic, commensal and MDR *E. coli* ST 155 carrying distinct AMR markers, which may be related to previous evidence of efficient transference of resistance features to pathogenic *E. coli* strains via conjugation, favoring epidemiological success of resistant isolates [16,73].

The *E. coli* ST224 is an international high-risk clone, which presents a high genetic plasticity, enabling its pandemic dissemination and adaptation to several ecosystems. Indeed, clinical, animal, and environmental ST224 strains have been commonly associated with MDR isolates harboring a wide range of resistance mechanisms, including *bla*_NDM_, *bla*_CTX-M_, *bla*_KPC_, *mcr-1,* and *fosA* genes in China, Pakistan, Brazil, Spain, India, Egypt, and Thailand [2,71,74,75,76,77,78,79,80]. Interestingly, most of *E. coli* ST224 were recovered from wild fish, found to be genetically related (PFGE—subcluster C1) and susceptible to the tested antibiotics, suggesting a low/limited anthropogenic and selective pressure at OPQ, a location with difficult access in the region and more remote compared to other *A. gigas* capture and farming sites.

The international high-risk clone ST1196 plays a critical role in the expansion of multiple AMR markers among *E. coli* isolates from distinct settings, leading to the emergence of heterogenous antibiotic resistance phenotypes, including a strong association with colistin, third generation cephalosporins, and aminoglycoside-resistant isolates. Among animal-related samples, this ST has been highly associated with *E. coli* in poultry samples from Germany, Czech Republic, Thailand, and Egypt, along with the carriage of AMR genes variants, such as *bla*_TEM_, *bla*_CTX-m_, *bla*_CYM_, *bla*_NDM_ and *mcr* [56,79,81,82]. In Spain, it was detected in high prevalence in waste-water treatment plants among genetically related strains co-harboring rmtB, *bla*_CTX-m-55_ and *mcr-1* genes [2]. In clinical settings, it has been responsible for the dissemination of KPC-2- and NDM-1-producing isolates in Pakistan hospitals [45]. Diverging from previous reports, ST1196 was related only with a highly susceptible isolate from F4, suggesting initial spread of this clone and/or selective pressure against it due to the presence of resistant strains.

The *E. coli* ST226, which was related with a CTX-M-producing MDR isolate at F4, has been previously linked with *bla*_CTX-M_ variants among different animal hosts, including humans. In Egypt and Portugal, this genotype was detected in human, beef and bovine isolates presenting *bla*_CTX-M-1_, *bla*_CTX-M-32_ and *bla*_CTX-M-2_ genes, respectively, and in China where *bla*_oxa-1_ was harbored among farmed minks [78,82,83,84]. In addition, human fecal carriage in Portugal and Cambodia, and a patient with MCR-1-producing isolates causing acute diarrhea in Thailand, were also reported [85,86,87].

There are few reports of *E. coli* ST1249, which has been described as ESBL- and MCR-producing *E. coli* among food items in the Netherlands, diseased chicken in the Czech Republic, and companion animals (dogs and cats) in Europe [88,89,90,91]. This clone was restricted to F2 among genetically related isolates (subcluster A2) harboring the *qnrA* gene, also being the first report in Brazil of this clone among animal samples.

There are limited data regarding the prevalence *E. coli* ST949, which is known for its pathogenic potential due to a wide range of virulence capabilities [12]. Recent evidence indicates it is an emerging clone with pathogenic potential that could mainly spread through water sources, even though it has been reported as causing infection in animals and humans [92,93,94,95]. This ST was curiously related to a commensal *E. coli* isolate from wild fish at MZG, contrary to the evidence of virulence potential, but also pointing to its emergence as it was first reported in Brazil and has the potential of dissemination as genetically revealed by PFGE (subcluster A2).

The few reports of ST1431 are from different sources, and mostly related with ESBL- and MCR-producing isolates from different sources, including *E. coli* isolates harboring *bla*_CTX-M_ detected in gulls in Canada and Chile, and wastewater in Algeria, along with MCR-producing isolates in raw meat and farmed rabbits in Portugal [96,97,98]. Curiously, this ST was related with a highly resistant CTX-M-producing and susceptible isolate at F4, suggesting a possible acquisition and transmission potential of genetic elements containing *bla*_CTX-M_ variants.

Rarely reported clones, such as ST4380 and ST4482, were found firstly in Brazil. ST4380 was previously related to ESBL-producing isolates from animal samples (European starlings in USA and monkeys in France), while ST4482 was related to uropathogenic *E. coli* (UPEC) strains in Pakistan and quinolone-resistant strains from poultry in the Philippines [99,100,101,102]. Both STs were detected in MZG, however only ST4482 was associated with a highly resistant CTX-M-producing isolate.

The PFGE typing allowed deeper insights into the remarkable genetic diversity of *E. coli* isolates, demonstrating genetic relatedness among strains from distinct sources (wild and farmed fish) as observed in subclusters A2, A3, B1, B2 and C2, which might be explained by the fact that many fish farmers capture *A. gigas* matrices in the natural environment to start breeding and production. Yet, genetically similar isolated (subclusters A1 and C1) in the same locations were verified, suggesting selection and clonal expansion of strains within a localized site. Moreover, differences on the phenotypical and molecular resistance features of isolates belonging to the same cluster (clone) suggest the impact and dissemination of mobile genetic elements containing *bla*_CTX-M_, *qnrA* and *qnrB* genes in this region. Previous reports of extensive genetic diversity among *E. coli* isolates in seafood from Brazil and the United States, as well as data on the present study, corroborate the need for molecular typing and genetic relatedness investigations on the surveillance of *E. coli* strains from different natural hosts, sources, and locations, as their critical role in transmission of AMR characteristics [103].

## 5. Conclusions

The present study demonstrated a high genetic background among quinolone-resistant *E. coli* isolates in *A. gigas*, revealing that this fish species could act as bioindicators of clinically significant AMR genes in aquatic niches and food-producing animals, even in poorly explored environments with low antimicrobial drugs exposure, in the Brazilian Amazon region. International high-risk clones and genetically related strains were associated with ESBL and PMQR genes (*bl*a_CTX-M_, *qnrA* and *qnrB*), suggesting a multiclonal spread of *E. coli* strains harboring AMR genes in distinct genetic contexts, and highlight the importance of molecular typing studies for surveillance of potential threatening strains. Finally, this study underscores the emergence, spread and interference of AMR bacteria in wildlife and environmental matrices in the vicinity of anthropogenic environments.

## Figures and Tables

**Table 1:** Antimicrobial susceptibility characteristics of *E. coli* isolates from farmed and wild *A. gigas* in the Brazilian Amazon.

Antimicrobial	Farmed (*n* = 16)		Wild * (*n* = 18)	
	S (%)	NS (%)	S (%)	NS * (%)
AMP	4 (25)	12 (75.0)	3 (16.6)	15 (83.4)
SAM	5 (31.25)	11 (68.75)	7 (38.9)	11 (61.1)
TZP	14 (87.25)	2 (12.75)	18 (100)	0
CXM	4 (25)	12 (75.0)	6 (33.3)	12 (66.7)
FOX	11 (68.75)	5 (31.25)	7 (38.9)	11 (61.1)
CAZ	13 (81.25)	3 (18.75)	8 (44.4)	10 (55.6)
CRO	12 (75)	4 (25.0)	6 (33.3)	12 (66.7)
FEP	15 (93.75)	1 (6.25)	7 (38.9)	11 (61.1)
CIP	0	16 (100.0)	0	18 (100.0)

S: susceptive; NS: non-susceptive (intermediate and resistant isolates). * Higher frequency of NS isolates (*p* = 0.005).

**Table 2:** Phenotypical and molecular resistance features of *E. coli* strains isolated from *Arapaima gigas* in the Brazilian Amazon.

Sample	Source	Resistance Profile	MDR	Molecular Profile	Virulence	ST	PFGE
01	F1	AMP, CIP	−	*qnr*A	Commensal	448	D
02	F1	AMP, SAM, CRX, CFO, CIP	+	*bla*_CTX-M_	Commensal	448	A1
04	F1	AMP, CIP	−	*qnr*B	Commensal	448	A1
06	F1	AMP, CIP	−	*qnr*B	Commensal	448	A1
07	F2	AMP, SAM, CIP	+	*qnr*A	Commensal	1249	A2
08	F2	AMP, SAM, CIP	+	*qnr*A	Commensal	1249	A2
09	F2	AMP, SAM, CIP	+		Commensal	7973	B2
10	F2	AMP, CIP	−		Commensal	1249	NT
12	OPQ	AMP, CIP	−		Commensal	224	C1
14	OPQ	AMP, CIP	−		Commensal	224	C1
16	OPQ	CIP	−		Commensal	224	C1
17	OPQ	CIP	−		Commensal	224	C1
18	CUT	CIP	−	*qnr*A	Commensal	155	B1
19	F3	AMP, SAM, CRX, CFO, CRO, CIP	+	*bla*_CTX-M_	ETEC	NT	B1
20	F3	AMP, SAM, CRX, CFO, CRO, CIP	+	*bla*_CTX-M_	ETEC	155	NT
21	F3	CIP	−	*qnr*A	ETEC	155	B1
27	MZG	AMP, SAM, CIP	+		Commensal	4380	A3
28	MZG	AMP, SAM, CIP	+		Commensal	949	A2
30	MZG	AMP, CRX, CFO, CRO, CIP	+		Commensal	NT	NT
31	MZG	AMP, CRX, CFO, CAZ, CRO, CPM, CIP	+	*bla*_CTX-M_	Commensal	4482	B
33	MZG	AMP, CRX, CFO, CAZ, CRO, CPM, CIP	+	*bla*_CTX-M_	Commensal	NT	B1
45	F4	AMP, SAM, PPT, CRX, CFO, CAZ, CRO, CPM, CIP	+	*bla*_CTX-M_	Commensal	226	C2
46	F4	AMP, SAM, PPT, CRX, CFO, CAZ, CRO, CIP	+	*bla*_CTX-M_	Commensal	1431	A3
47	F4	CIP	−		Commensal	224	B2
48	F4	CIP	−		Commensal	1196	B1
49	F4	CIP	−		Commensal	1431	A3
50	TT	AMP, CRX, CFO, CAZ, CRO, CPM, CIP	+	*bla*_CTX-M_	Commensal	7973	C2
51	TT	AMP, CRX, CFO, CAZ, CRO, CPM, CIP	+	*bla*_CTX-M_	Commensal	7973	C2
52	TT	AMP, CRX, CFO, CAZ, CRO, CPM, CIP	+	*bla*_CTX-M_	Commensal	7973	C2
53	TT	AMP, CRX, CFO, CAZ, CRO, CPM, CIP	+	*bla*_CTX-M_	Commensal	7973	C2
54	TT	AMP, CRX, CFO, CAZ, CRO, CPM, CIP	+	*bla*_CTX-M_	Commensal	7973	C2
56	TT	AMP, CRX, CFO, CAZ, CRO, CPM, CIP	+	*bla*_CTX-M_	Commensal	7973	C2
66	FG	AMP, SAM, CRX, CFO, CAZ, CRO, CPM, CIP	+	*bla*_CTX-M_	Commensal	7973	C2
67	FG	AMP, SAM, CRX, CFO, CAZ, CRO, CPM, CIP	+	*bla*_CTX-M_	Commensal	NT	NT

AMP: Ampicillin, SAM: Ampicillin/Sulbactan, PPT: Piperacillin/Tazobactan, CRX: Cefuroxime, CFO: Cefoxitin, CAZ: Ceftazidime, CRO: Ceftriaxone, CPM: Cefepime, CIP: Ciprofloxacin, OPQ: Oiapoque, CUT: Cutias, MZG: Mazagão, TT: Tartarugalzinho, FG: Ferreira Gomes, ST: sequence type.

## Data Availability

All relevant data are presented within the manuscript.
